# A REVIEW OF PAIN MANAGEMENT FOR PEOPLE WITH DEMENTIA: A GENERAL SURGICAL PERSPECTIVE

**DOI:** 10.1093/geroni/igad104.3149

**Published:** 2023-12-21

**Authors:** Emily Neville, Ruth Wagstaff, Snezana Stolic, Christine Neville

**Affiliations:** University of Notre Dame Australia, Wagga Wagga, New South Wales, Australia; Charles Darwin University, Darwin, Northern Territory, Australia; University of Southern Queensland, Toowoomba, Queensland, Australia; University of Southern Queensland, Toowoomba, Queensland, Australia

## Abstract

As the global population ages and the prevalence of dementia increases, more people with dementia will present for surgery. Perioperative pain management, particularly for people who are not able to communicate effectively, is challenging for clinicians. Poor surgical outcomes may result if pain is not managed appropriately. The objective of this review was to synthesize the existing research about how pain is managed for people with dementia in the perioperative setting, discuss the implications for general surgery so clinical practice and surgical outcomes are improved. After an extensive database search, the themes identified were pain assessment including commonly used rating scales, the types and amount of pain relief, that people with dementia received less analgesia than people without dementia and the challenges for effective pain management. Findings determined that pain management needs a stronger evidence base, challenges are due to the impaired ability of the person with dementia to communicate pain and that clinicians have difficulty understanding pain behavior in people with dementia. Adequate pain management for people with dementia in the perioperative setting is important for a faster and better recovery and this matter will only become a bigger issue as the ageing population increases.

